# HIV-1 Protease and Reverse Transcriptase Control the Architecture of Their Nucleocapsid Partner

**DOI:** 10.1371/journal.pone.0000669

**Published:** 2007-08-22

**Authors:** Gilles Mirambeau, Sébastien Lyonnais, Dominique Coulaud, Laurence Hameau, Sophie Lafosse, Josette Jeusset, Isabelle Borde, Michèle Reboud-Ravaux, Tobias Restle, Robert J. Gorelick, Eric Le Cam

**Affiliations:** 1 Laboratoire de Microscopie Moléculaire, UMR 8126: Interactions moléculaires et cancer, CNRS, Université Paris Sud-Institut de Cancérologie Gustave Roussy, Villejuif, France; 2 Division de Biochimie, UFR des Sciences de la Vie, Université Pierre et Marie Curie-Paris, Paris, France; 3 Laboratoire Biologie et Multimedia, Université Pierre et Marie Curie-Paris, Paris, France; 4 Laboratoire d'Enzymologie Moléculaire et Fonctionnelle, CNRS FRE 2852, Institut Jacques Monod, CNRS-Université Pierre et Marie Curie-Paris, Paris, France; 5 Institut für Molekulare Medizin, Universitätsklinikum Schleswig-Holstein and ZMSB, Lübeck, Germany; 6 AIDS Vaccine Program, Basic Research Program, Science Applications International Corporation at Frederick, The National Cancer Institute at Frederick, Frederick, Maryland, United States of America; Vanderbilt University, United States of America

## Abstract

The HIV-1 nucleocapsid is formed during protease (PR)-directed viral maturation, and is transformed into pre-integration complexes following reverse transcription in the cytoplasm of the infected cell. Here, we report a detailed transmission electron microscopy analysis of the impact of HIV-1 PR and reverse transcriptase (RT) on nucleocapsid plasticity, using in vitro reconstitutions. After binding to nucleic acids, NCp15, a proteolytic intermediate of nucleocapsid protein (NC), was processed at its C-terminus by PR, yielding premature NC (NCp9) followed by mature NC (NCp7), through the consecutive removal of p6 and p1. This allowed NC co-aggregation with its single-stranded nucleic-acid substrate. Examination of these co-aggregates for the ability of RT to catalyse reverse transcription showed an effective synthesis of double-stranded DNA that, remarkably, escaped from the aggregates more efficiently with NCp7 than with NCp9. These data offer a compelling explanation for results from previous virological studies that focused on i) Gag processing leading to nucleocapsid condensation, and ii) the disappearance of NCp7 from the HIV-1 pre-integration complexes. We propose that HIV-1 PR and RT, by controlling the nucleocapsid architecture during the steps of condensation and dismantling, engage in a successive nucleoprotein-remodelling process that spatiotemporally coordinates the pre-integration steps of HIV-1. Finally we suggest that nucleoprotein remodelling mechanisms are common features developed by mobile genetic elements to ensure successful replication.

## Introduction

The human immunodeficiency virus type I (HIV-1) encodes three enzymes: protease (PR), reverse transcriptase (RT) and integrase (IN). These facilitate the transfer of genetic information from the HIV-1 genome to the nuclear chromatin of the infected cell. The homodimeric PR drives the viral maturation process, by the sequential cleavage of the viral Gag and Gag-Pol precursors [1]. Within the maturing capsid, this process results in the collection of RT, IN, Vpr and the nucleocapsid, a condensed ribonucleoprotein complex predominantly comprised of viral RNA coated with mature nucleocapsid protein (NCp7) [2], [3]. Subsequently, within the confines of the cytoplasm of the infected cell, the heterodimeric RT catalyses by a sophisticated process the conversion of the RNA genome into double-stranded DNA (see Animation S1, S2 for an animated and detailed model of reverse transcription). Finally, IN (presumably in a tetrameric form [4]) catalyses the concerted insertion of the two viral DNA ends into the cell's chromosomal DNA, allowing the viral DNA or provirus to behave as a cellular genetic unit.

A unique intracellular pathway involving actin, microtubules and nuclear pores results in the reverse transcribed HIV-1 genome, which is incorporated into a pre-integration complex (PIC) that is actively transported into the nuclei of non-dividing cells [5]. The molecular events that define this route remain to be clarified. However, this process requires a progressive remodelling of the viral nucleoprotein architecture. Three critical steps can be delineated at this level: i) the formation of a condensed RNA-associated nucleocapsid within the mature capsid [2]; ii) capsid uncoating [6] and iii) the conversion to a PIC [7]. The related molecular mechanisms require clarification, and also raise questions regarding the plasticity of the nucleocapsid during HIV-1 maturation and reverse transcription.

The HIV-1 nucleocapsid architecture remains poorly defined. Within mature HIV-1 cores, viral RNA and NCp7 are located within a condensed ribonucleoprotein complex that also contains other viral (RT, IN, Vpr, Nef [8]) and cellular components, one of which is tRNA^Lys3^ [9]. NCp7 (55 aa.) is a basic and flexible peptide, with a critical N-terminal basic domain and two highly structured and conserved zinc-finger domains separated by a proline-rich linker [10]. In capsid-free systems, aggregates have been observed with NCp7 or NCp9 (the ultimate NCp7 precursor), tRNA^Lys3^ [11], RNA [12], as well as DNA [13]. These *in vitro* co-aggregation properties and their modulation have been proposed to relate directly to the nucleocapsid architecture and its plasticity [14]. The aggregates form at the micromolar level with an NCp:occluded-site stoichiometric ratio of 1∶1 [15], a value close to that observed in virions. Spherical particles containing hundreds of nucleic acid molecules are observed via transmission electron microscopy (TEM) to condense and co-aggregate with thousands of NCp molecules in a complex and dense nucleoprotein framework [12], [14]. Both the basic residues and the zinc-finger structures of NCp7 are required for a potent effect, while some NCp7-NCp7 protein contacts directed by nucleic acid binding are also suspected to be involved within these complexes [14]. In addition, recent findings [16] demonstrated that NCp7 can bridge between non-contiguous regions of nucleic acid oligomers, which could account for part of NCp7′s aggregative properties. Interestingly, with magnesium-containing buffers that facilitate efficient RT activity, co-aggregation is strongly reduced with double-stranded DNA (dsDNA). This effect is observed with the mature NCp (NCp7), but not its precursor NCp9 [14]. On the other hand, NCp15 is deficient in aggregating both single-stranded DNA (ssDNA) and dsDNA, resulting in DNA coating similar to prototypical single-stranded binding (SSB) proteins [14], [17].

During viral maturation, nucleocapsid condensation and stabilization of dimeric viral RNA require the complete processing of NC [3], [18], [19]. Physical cleavage of the capsid (CAp24) and nucleocapsid (NCp7) proteins from their Gag precursor (MA-CA-p2-NC-p1-p6) occurs via the primary proteolytic cleavage p2-NC, catalysed by PR, which leads to NC-p1-p6 (NCp15) [2]. p6 removal from NCp15 results in a transient accumulation of NCp9 (NCp7-p1) followed by liberation of NCp7 and p1 at the last proteolysis step. The corresponding PR cleavage sites are considered as secondary and tertiary cleavage site classes, although RNA strongly activates the processing of NCp15-to-NCp7 *in vitro* [1], [20]. Moreover, mutations within both PR-cleavage sites, especially the p1-p6 site, favour resistance against certain protease inhibitors [21], [22], highlighting that NCp15 processing is essential for viral infectivity.

Reverse transcription complexes (RTCs) evolve from within the nucleocapsid [23]. As a nucleic acid chaperone protein, NCp7 efficiently assists RT in defined critical events [24] (described in the Animation S1/S2) such as tRNA^Lys3^-directed initiation, the two obligate strand transfers and central DNA flap termination. Stability of the newly synthesized HIV-1 DNA within the cytoplasm of infected cells also requires the presence of functional NCp7, as viral DNA instability has been shown in HIV-1 mutants with altered N-terminal or zinc finger domains [25]. In contrast, NCp7 as well as CAp24 appear predominantly to dissociate from the full-length linear viral dsDNA extracted from infected cells within purified RTCs/PICs [7], [26], [27]. The apparent loss of NCp7 in contrast to the efficient retention of IN and Vpr (viral protein R) within RTCs and PICs suggests that nucleocapsid disassembly and PIC assembly are directly interconnected during the reverse transcription process, while capsid uncoating is also critical for the generation of an active PIC [6].

A previous study, imaging uranyl-stained nucleoprotein complexes with TEM in annular darkfield mode, showed a selective preference for NCp7 to co-aggregate with RNA and ssDNA [14]. Taking this into account, we address the architectural changes in the property of HIV-1 NCp complexed with DNA in the context of PR and RT activity. The C-terminal cleavage of NCp15 by PR is identified as the essential step that liberates NCp7 for HIV-1 nucleocapsid condensation. Furthermore, the later double-stranded DNA synthesis by RT appears to be the key step for HIV-1 nucleocapsid protein removal.

## Results

### NCp15 processing by PR requires single-stranded nucleic acids to generate NCp7, a reaction that results in nucleoprotein aggregation

**Figure 1:**
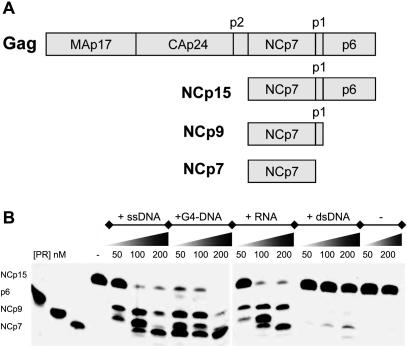
Proteolysis of NCp15 by PR. (A) Sequential and ordered proteolysis of the HIV-1 NC domain. Digestion of the Gag polyprotein by PR leads to the mature NCp7 via the NCp15 and NCp9 intermediate forms [1]. (B) Proteolysis of NCp15 (1 µM) at 30°C and pH 6.6 for 2 h. The products shown were generated using three concentrations of recombinant PR (50, 100 and 200 nM) and incubated in the presence of a circular ssDNA (3,352 nt, 1.25 nM), a G4-DNA (250 nM), a 415-mer RNA fragment corresponding to the HIV-1 leader region (20 nM) or a 3,352 dsDNA plasmid (1 nM). Controls: p6, NCp9 and NCp7 are shown in the three far left lanes, whereas products of NCp15 incubation with two concentrations of PR (50 and 200 nM) in the absence of nucleic acid are shown to their right.

*In vitro* processing of NCp15 (1 µM) by PR (Figure 1) was studied by examining the effect of several nucleic acids (ssDNA, G4-DNA, RNA and dsDNA) in order to address the positive effect on proteolysis, already shown for RNA [18], [20]. Using three PR concentrations resulting in a NCp15-to-PR molar ratio of 20, 10 and 5 respectively, we found that activation of NCp15 proteolysis in the presence of nucleic acids effectively depended on their nature. With dsDNA a very weak stimulation was observed, whereas in the absence of nucleic acids PR was not active in processing NCp15 under our assay conditions. With ssDNA or RNA the digestion profiles were much more prominent, and clearly showed that NCp9 was an obligate intermediary. G4-DNA was the most efficient cofactor in promoting the C-terminal processing of NCp15. With its four short single-stranded 3′ tails linked to six stacked G-quartets [28], this small DNA molecule has a high affinity binding site for NCp7 and NCp9 [29]. Thus strongly bound NCp15 species appear to be the best substrates for complete processing.

**Figure 2:**
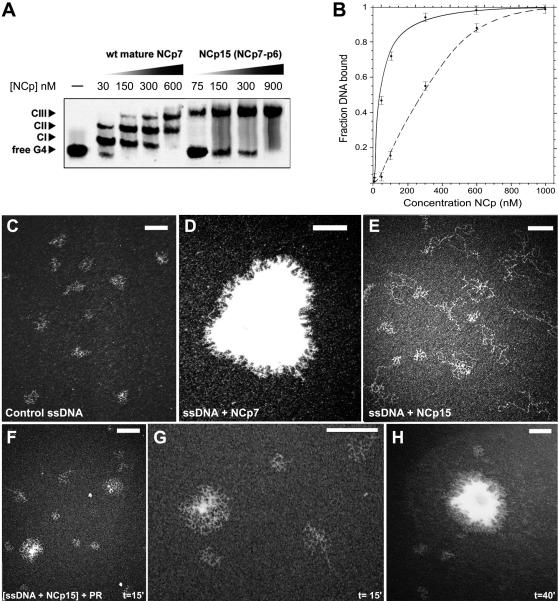
Differences in DNA binding between NCp15 and NCp7 and the effect of NCp15 proteolysis. (A,B) EMSA using G4-DNA (5 nM) and increasing amounts of NCp7 or NCp15. Complexes were formed for 15 min. at 37°C before electrophoresis. Note that in the case of NCp15 there is only one shifted G4-DNA species (A), whereas quantification of the DNA bound fraction as a function of the total NCp concentration (B) shows a 6-fold drop in affinity for G4-DNA with NCp15 (solid line) compared to NCp7 (dashed line). (C,D,E) TEM visualization of ssDNA (5 nM) without protein (C) or with saturating amounts (3.4 µM) of NCp7 (D) or NCp15 (E). (F,G,H) TEM visualization of NCp15 proteolysis in the presence of ssDNA after 15 min. (F and G) and after 40 min. (H) incubation with saturating amounts of NCp bound to ssDNA and a NCp15-to-PR ratio of 30 corresponding to [NCp15] = 3.4 µM, [PR] = 100 nM and [ssDNA] = 5 nM. The nascent aggregates revealed in (F) are shown with a higher magnification in (G). Scale bars in each panel correspond to 150 nm.

The affinity of NCp15 for G4-DNA (10 nM) was examined and compared with NCp7 in a gel shift assay (Figure 2 A–B). NCp7 formed three species with G4-DNA with the first complex showing an apparent K_d_ near the nM range. NCp15 formed only one detectable complex with a six-fold weaker affinity. NCp15 was also unable to co-aggregate with ssDNA, unlike NCp7, as shown by TEM visualization (Figure 2 C–E). Instead NCp15 covered ssDNA like a SSB protein, spreading the DNA on the carbon film [14]. In contrast, NCp7 strongly co-aggregated with RNA and ssDNA (TEM, Figure 2D and [14]). Thus interactions achieved with NCp7 for both the co-aggregation with ssDNA and the multiple binding with G4-DNA were not possible with NCp15. Examination of NCp9 in both assays showed that its behaviour was similar to NCp7 but with a stronger aggregative activity [14], [29], suggesting that p6 inhibits the aggregative properties of the NC domain within NCp15.

We next followed the aggregation of the ssDNA that occurs after processing of NCp15 by PR with TEM visualization (Figure 2F–H). PR-cleavage of NCp15 was indeed able to overcome the p6-mediated inhibition during the course of ssDNA-activated proteolysis. At 15 min., an intermediary step could be visualized via NCp15 conversion to NCp9 and NCp7 species with a limited number of aggregated ssDNA surrounded by some NCp15-coated ssDNA (Figure 2 F,G). At 40 min., prototypical NCp7- or NCp9-ssDNA co-aggregates were visible (Figure 2H) under conditions where a mixture of NCp9 and NCp7 were produced. Such a reaction clearly mimics the nucleocapsid condensation within the virion.

### From ssDNA-NCp7 co-aggregates, dsDNA extrudes and escapes during HIV-1 RT-directed DNA synthesis, whereas NCp7 remains bound to the central DNA flap

**Figure 3:**
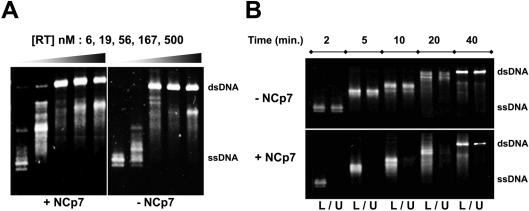
Comparison of DNA synthesis by RT in the absence and presence of NCp7. (A) The progression of DNA synthesis is shown with or without NCp7 for a reaction time of 30 min. as a function of RT concentration. (B) The reaction is shown as a function of time with or without NCp7 for a fixed concentration of RT. The DNA products (B) have been sedimented before electrophoresis. Lower (L) and upper (U) half-volumes collected after sedimentation are indicated underneath. Concentrations of ssDNA and NCp were, respectively, 5 nM, 3.4 µM (A and B) and RT was 50 nM (B). DNA and NCp7 were premixed for 4 min. at 37°C, following incubation with RT for 2 min. before addition of dNTPs (100 µM each) to start the reaction.

The efficiency of reverse transcription within NCp7–ssDNA aggregates was addressed using an *in vitro* assay of DNA synthesis with a circular recombinant ssDNA template encompassing the minus DNA strand of the HIV-1 cPPT/CTS locus. Adding NCp7 to the assay improved the 99 nt-long strand displacement synthesis required at the end of the reaction to generate the HIV-1 central DNA flap [30]. DNA synthesis along the ssDNA template was not critically affected when saturating amounts of NCp7 (1 NCp7 *per* 5 nucleotides) were added with increasing amounts of RT (from 6 nM to 500 nM), as shown after an electrophoresis of the deproteinized DNA products (Figure 3A). A more detailed examination suggested that adding NCp7 apparently allowed a slightly more processive elongation for the lowest RT concentration. Using RT in large excess, some degraded dsDNA products were observed, due to some contaminating nuclease(s). Under these conditions (5 mM magnesium and 50 mM sodium in the absence of RT), NCp7 is known to co-aggregate strongly with ssDNA, but very poorly with dsDNA [14]. The behaviour of the DNA circles during the course of DNA synthesis was examined by sedimentation and electrophoresis (Figure. 3B). We fixed the RT concentration at an intermediate level (50 nM with 6 nM of primed DNA) sufficient to catalyse an almost complete DNA synthesis within 30 min., with or without NCp7. Electrophoresis revealed that in the presence of NCp7, DNA molecules were fully aggregated during the course of the reaction without significantly affecting DNA polymerisation, whereas a significant portion of final DNA products could be recovered as soluble circles.

**Figure 4:**
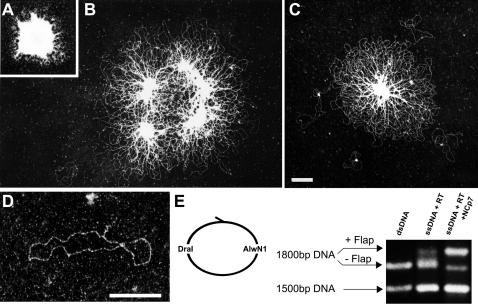
Extrusion of dsDNA produced by RT from ssDNA-NCp7 co-aggregates. Progression of DNA synthesis analyzed by TEM after 2 min. (A), 10 min. (B) and 40 min. (C) from reactions with ssDNA (5 nM), RT (50 nM) and NCp7 (3.4 µM). Disaggregation after 10 min. (B) appeared both at the periphery and within the aggregates. A few individual molecules were visible close to the aggregate after 40 min. (C). (D) A typical DNA product visualized by TEM after 40 min. of DNA synthesis with subsequent incubation for 15 min. at 70°C in the presence of 0.4 M NaCl. (E) Band shift analysis of DNA flap synthesis within the dsDNA produced by RT after 40 min. at 37°C with 50 nM RT, with or without 3.4 µM NCp7. An excess of DraI and AlwnI enzymes that digest this dsDNA into two fragments (1800 and 1500 bp) was added. The DraI-AlwnI digestion products of the plasmid DNA are shown as a control on the left. When the HIV-1 central DNA flap is fully synthesized (i. e. with NCp7), the 1800 bp fragment is shifted to a slower migrating band. Magnification is identical for panels A,B,C. The scale bars correspond to 250 nm.

Progression of DNA synthesis in the presence of NCp7 was therefore visualized directly with TEM after incubation for 2, 20 and 40 min. (Figure 4 A,B,C respectively). The corresponding micrographs provide convincing evidence that elegantly depicts the reduction in aggregation during the DNA synthesis. At 2 min., dsDNA still comprised a very minor portion of the total nucleic acids and TEM revealed that all DNA circles were still highly aggregated in globular particles (Figure 4A), with a similar morphology compared to the NCp7-ssDNA in the absence of RT (Figure 2D). Once dsDNA was formed it was released from the aggregates. At 20 min., dsDNA represented roughly one half of the DNA circles. The aggregates were partially relaxed and extended. The disaggregated dsDNA was visible at the periphery as well as between the residual aggregates (Figure 4B). At 40 min., reduced networks with only small compact centres, as well as individual molecules were observed (Figure 4C). The dsDNA circles that separated from the networks contained a residual electron-dense aggregate that corresponded to central DNA flaps bound by NCp7 (see also [14]). Indeed, the addition of 0.4 M NaCl to the samples with 10 min. of incubation at 70°C prior to depositing on grids revealed the flap. The residual globular networks of NCp7 were disrupted, but a small piece of stretched DNA extended from the circles. This surprising finding suggested that the NCp7 network bound to the DNA flap was rearranged during the treatment in a way that allowed ssDNA to spread like a canonical SSB protein (Figure 4D). Moreover, in accord with our previous study [30], most of the DNA products contained a complete central DNA flap (Figure 4E). Their DraIII-AlwNI digestion generated a discrete band shift indicative of the longer linear fragment (1800 bp), containing the flap structure, in contrast to the same fragment obtained after digestion of the corresponding dsDNA plasmid (control without a DNA flap) or of RT-generated dsDNA without NCp7 (heterogeneous flap and broader distribution of products).

### NCp-ssDNA co-aggregation followed by nascent dsDNA extrusion characterizes the wild-type HIV-1 NCp7-RT pair

In order to better understand the critical NCp determinants involved in ssDNA aggregation and/or in dsDNA extrusion, several NCp forms were compared in RT assays and examined after centrifugation or direct TEM visualization of DNA products (Figure S1). The corresponding results are commented in the Dataset S1, from which it can be concluded that the N-terminal domain and the zinc fingers of NCp7 are necessary to promote aggregation of ssDNA followed by controlled extrusion of dsDNA. Extension of the C-terminus of NCp7 (by the addition of p1, i.e. NCp9) appeared not to affect aggregation, but limited the apparent release of both dsDNA and NCp. Indeed, significantly stronger retention of dsDNA with ssDNA-NCp9 co-aggregates in Mg^2+^-containing buffer has already been shown [14], whereas in force-induced DNA denaturation experiments, NCp7 has been proposed to have an increased mobility when bound to DNA compared to NCp9 [17].

As HIV-1 RT appeared not to be active in the presence of Moloney-murine leukaemia virus (Mo-MuLV) NCp10, we tested whether Mo-MuLV RT was active under these conditions (Figure S1 and Dataset S1). Mixing Mo-MuLV RT with Mo-MuLV NCp or HIV-1 NCp in the assay led to efficient DNA synthesis. Therefore, Mo-MuLV NCp10 does not complement HIV-1 RT, whereas HIV-1 NCp7 does so for Mo-MuLV RT.

## Discussion

The present results illustrate how a nucleoprotein architecture that mimics a retroviral nucleocapsid can be directly modulated by two retroviral enzymes: i) a protease, PR, that preferentially cleaves its NCp substrate once bound to nucleic acids, thus changing its binding properties to a strongly aggregative mode; and ii) a DNA polymerase, RT, which translocates along its single-stranded template, changing it to a more weakly aggregative double-stranded nucleic acid form while displacing the initially bound NCp7 proteins. With such remarkable remodelling properties, demonstrated here for the first time, it is clear that HIV-1 PR and RT are engaged in controlling the nucleoprotein architecture of the HIV-1 genetic material between viral maturation and cellular infection. Below, we discuss the biological impact of these findings in the context of the HIV-1 replication cycle.

**Figure 5:**
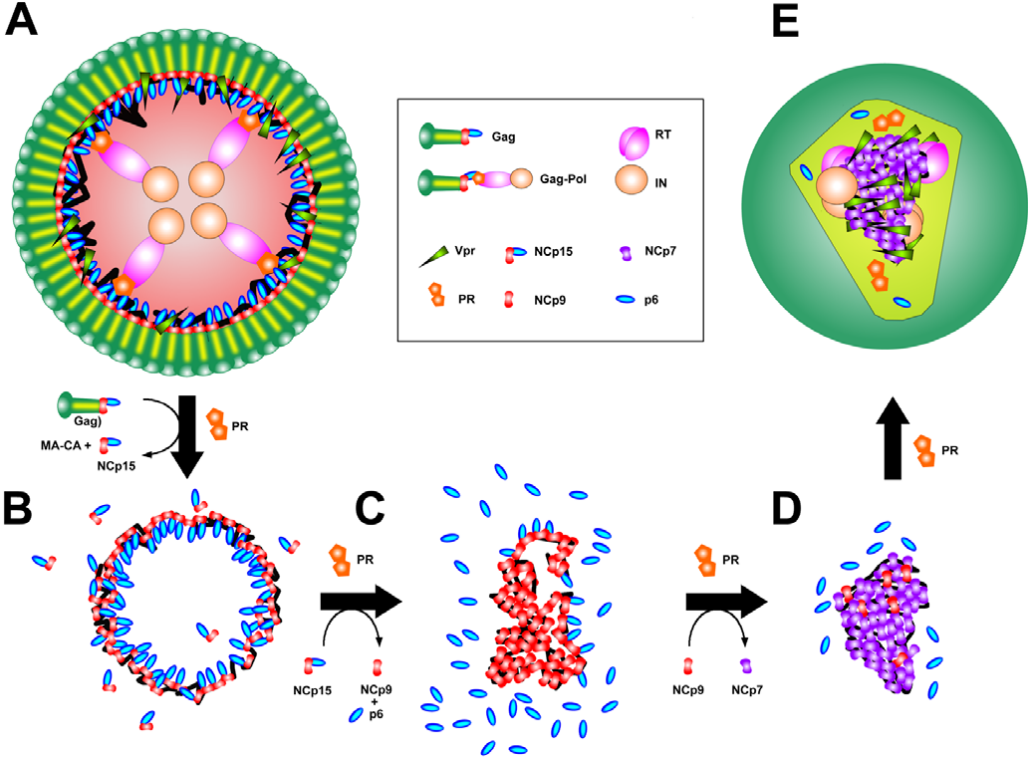
Hypothetical model of HIV-1 nucleocapsid assembly controlled by PR-directed NCp15 proteolysis. The HIV-1 immature (A) and mature (E) capsid are depicted, focusing on the nucleoprotein region. This model was inspired from L. Henderson's (NCI-Frederick) illustration, taking into account the ratios of Gag, Gag-pol, NCp, PR, RT, IN and Vpr. (B) Gag cleavage between p2 and NCp15 by PR resulting in RNA-bound NCp15 removal from the capsid domains. (C) and (D) digestion of RNA-bound NCp15 yielding NCp9 (C), followed by NCp7 (D) which yields to an overall aggregation/condensation of the nucleocapsid that leads to (E).

Examining the proteolytic process whereby NCp7 is produced from RNA-bound NCp15, several conclusions can be drawn from our data, confirming the earlier study of Sheng et al. [18], [20]. A simplified model simulating the nucleocapsid maturation from an immature capsid is shown in Figure 5. NC domains in the Gag precursor (from ∼1500 copies [31] to 5000 [32]) cover the inner surface of the immature capsid (Figure 5A). These domains capture RNA and contribute to its shape with a weak RNA-chaperone activity [17]. The first event in Gag processing is PR cleavage between p2 and NCp15, which allows an overall physical separation of the NCp-RNA complex from the surrounding capsid [2] (Figure 5B). Once NCp15 is produced, two populations of NCp15 can be distinguished: one bound to RNA, fully covering it, and the other free or bound to non-RNA components. The NCp15 subpopulation that is not bound to RNA is not processed by PR (as shown in Figure 1A), unless other ligands stimulate PR activity. This subpopulation should be excluded from the final nucleocapsid architecture simultaneously with the fractional exclusion proposed for CAp24 from the conical network during capsid maturation [32], [33]. In contrast, the RNA-bound NCp15 pool is subjected to a new round of proteolysis that leads to NCp9, and then to an ultimate round that forms NCp7 [20] (Figure 5 C,D). Moreover, compensatory mutations are shown to emerge at the p1- p6 cleavage site to support drug-resistant PR [21], or directly to confer resistance against a new PR inhibitor [22]. p6 removal allows co-aggregation of NCp9 with nucleic acids (Figure 2, [14]). The subsequent p1 removal produced NCp7 already aggregated with the condensed RNA in a 1∶1 stoichiometric ratio. Therefore NCp7-NCp7 contacts (i.e. weak interactions) and/or NC bridging between non-contiguous nucleic acid regions [16] must be engaged in the nucleocapsid architecture. Contacts induced between RNA-bound NCp7 by p6/p1 removal are also suggested by the difference in binding to the G4-DNA substrate: multiple and discrete for NCp7, but single and weaker for NCp15. Conversely, NCp9 binding to the G4-DNA substrate easily shifts to an aggregated state, unlike NCp7 [29], and it is proposed to interact with RNA more stably than NCp7 [17]. This can be correlated with easier RT-directed removal of NCp7 after conversion of the genome to dsDNA (compare Figures 4 and S1). These data emphasize the C-terminal proteolysis of NCp, catalysed by PR, as a finely optimized mechanism that controls the conformation and reactivity of NC domains to drive the formation of nucleocapsid. A first step of condensation is followed by a second step that enhances plasticity. Such a mechanism can be linked with the stabilization of the RNA dimer [34] and, perhaps, the conical assembly of the mature capsid [35], [36].

With regard to interactions with the nucleocapsid, Vpr, RT and IN are depicted in our model as bulky partners (Figure 5), based on the proposed stoichiometry within the virion [32]. Vpr is retained within the capsid due to its affinity for Gag-associated p6 [37] and/or NC domains [38]. p6 domains directly favour the retention of the Gag-Pol protein within the immature capsid [39]. Most of PR, RT and IN proteins are presumed to be already in their mature form when PR digests NCp15 molecules. The series of events that drives auto-activation of PR and maturation of RT and IN also follows a sequential and ordered pathway, beginning with the p2-NC cleavage [40]. The precise locations of RT and IN within the maturing capsid still need to be defined; their presence is important for HIV-1 RNA maturation [41]. Moreover, IN interacts with RT and promotes reverse transcription [42]. Our data involving RT (Figure 4) suggests that this enzyme alone can easily diffuse into the nucleocapsid as it is readily accessible to primers in NCp7-ssDNA co-aggregates. Of interest is that HIV-1 NCp7 cannot be substituted in our assay with Mo-MuLV NCp10, which fully inhibits HIV-1 RT. Encapsidation of other cellular and viral proteins may also be controlled by the nucleocapsid architecture. Some cellular proteins, such as actin [43], APOBEC3G [44], and tRNA^Lys3^-synthetase [9], bind to the HIV-1 NC domains. Among the viral auxiliary proteins, i) Vif (viral infectivity factor) is poorly detectable in the HIV-1 mature core, even though it binds cooperatively to the HIV-1 leader RNA [45], ii) Nef (negative factor) is known to be sequestered into the viral core [46], iii) a PR-cleaved form of Tat (transactivator of transcription) required to assist reverse transcription is also suspected of being sequestered [47]. The mode of sequestration or exclusion within the HIV-1 core for each of these components can now be examined in the context of NCp7-RNA or NCp7-ssDNA co-aggregation. The three possibilities are: internalisation within the nucleocapsid: retention at its periphery, or exclusion during the condensation process.

**Figure 6:**
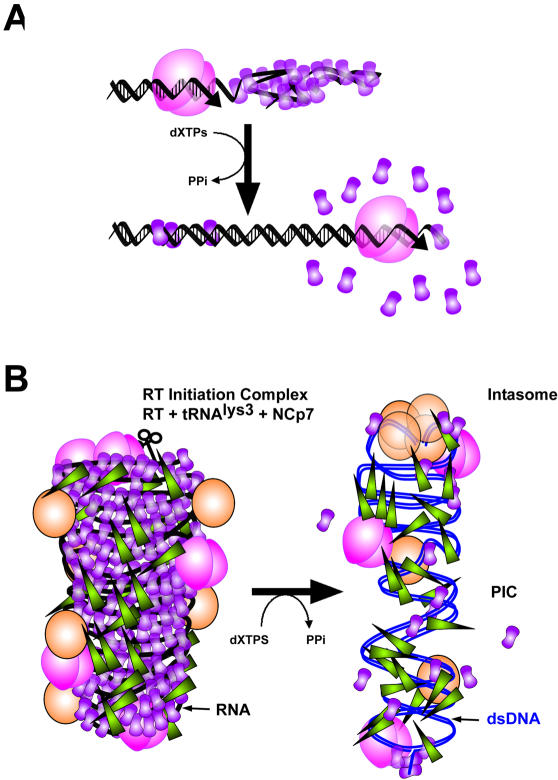
Hypothetical model for HIV-1 nucleocapsid dismantling controlled by RT directed dsDNA synthesis. (A) translocation of RT on a minus DNA template coated with NCp7. RT displaces NCp7 during this translocation reaction while converting ssDNA into dsDNA, which does not allow NCp7 to re-associate in an aggregative mode. The dismantling force is driven by dNTP hydrolysis that allows continuous RT translocation. Magnesium is critical as it weakens dsDNA-binding by NCp7. (B) macroscopic consequence of the NCp7 dismantling activity of RT. A reverse transcription complex is depicted from a perspective of the nucleocapsid architecture used in figure 5. Once reverse transcription is finished, resulting in dsDNA, the aggregative nucleic acid binding mode of NCp7 is drastically reduced, facilitating the binding of Vpr and IN (as well as other available cellular partners) and thus generating a PIC (represented here in a simplified way according to Nermut et al.[27]).

Examining DNA synthesis catalysed by RT in the presence of NCp7, the disruption of ssDNA-NCp aggregates is observed as ssDNA is converted into dsDNA. In Figure 6 another model is presented to show the remodelling activity of HIV-1 RT. With regard to DNA-directed DNA polymerisation (i.e. enzyme translocation along the ssDNA template to synthesize the dsDNA), the HIV-1 RT activity is almost as efficient with ssDNA aggregated with NCp7 as in an aqueous solution of naked ssDNA. Apart from DNA synthesis *per se*, the presence of magnesium, necessary for RT function, results in a strong and selective reduction of NCp-dsDNA aggregation due to a lower binding efficiency [14]. Under such conditions, the progressive disruption of the interactions involved in the initial NCp7-ssDNA co-aggregates demonstrates that NCp7 molecules bound to nucleic acids are progressively displaced by HIV-1 RT during its tracking along the minus strand DNA template, while re-binding of NCp7 to the dsDNA product is severely impaired (Figure 4). Hence HIV-1 RT acts here as a molecular motor that dismantles the overall nucleocapsid architecture, in a similar way to the dismantling of Rec-like nucleofilaments by certain DNA helicases [48]. In the case of RT, the energy is provided by dNTP hydrolysis, whereas the formation of dsDNA in the presence of magnesium robustly reduces NCp7 reassociation. This *in vitro* property strongly suggests that the formation of pre-integration complexes is a direct consequence of reverse transcription during the infection process. A complete separation of fully synthesized dsDNA from the residual aggregates is quite slow and may be assisted by other destabilizing factors. Finally, some residual NCp7 copies should, at the end of our assay, remain aggregated to the last single-stranded segment of the circular DNA, the central DNA flap, which is efficiently produced by RT with the assistance of NCp7 (Figure 4 D,E and [30]). These NCp7 molecules may contribute to the protection of the DNA flap structure and/or assist in its binding to yet to be identified cellular proteins.

Until now authentic changes of the nucleocapsid architecture have not been demonstrated during the process of reverse transcription, although NCp7 has been shown to assist RT in several critical steps [24]. Therefore, we speculate that two different modes might be involved in the disruption of this architecture (see Animation S1/S2). One consists of a progressive dismantling associated with RT translocation throughout the process. The other requires the formation of dsDNA, i.e. the plus strand DNA synthesis, taking into account that NCp7 should easily rebind to RNA-DNA heteroduplexes and to ssDNA, the transient species that appear during the process. Indeed, *in vitro* assays mimicking the minus strand synthesis have shown that NCp7 and the RNA-DNA/ssDNA products are aggregated [49], [50]. A subtle question arises as to what happens with the RNA fragments that are generated through the action of the RT-associated RNAseH during viral DNA synthesis [51], [52]. One would assume that they constitute an additional dispersing force for NCp7, causing its dissociation from RTCs, which may have occurred to a large extent at the time of plus strand DNA synthesis. RTC or PIC complexes isolated from infected cells contain IN and Vpr, but very little NCp7 [27]. For this reason we propose that NCp removal favours IN and Vpr sequestration on dsDNA (Figure 6B). Moreover, this dismantling may also favour the recruitment of cellular dsDNA binding proteins, with IN and Vpr possibly participating in the selection of suitable proteins. Such sequestrations and recruitments should be oriented and controlled during the plus strand DNA synthesis by binary initiation (3′ ppt and cppt) and termination (LTR duplication and central flap synthesis) [53]. Strands displaced during the course of termination should be the last portions to retain NCp7, especially the central DNA flap, while the HIV-1 LTR double-stranded DNA ends should promote active IN assemblies (Figure 6B).

Alongside this switch from nucleocapsid to PIC, there is another important point regarding architectural plasticity that concerns the relationships between reverse transcription, the nucleocapsid and the surrounding capsid assemblies. RTCs/PICs extracted from cells have lost not only NCp7, but also most of their CAp24 [26]. Yet the mechanism, as well as the spatiotemporal coordination of capsid uncoating are still not clearly understood [54]. It has been proposed that capsid uncoating is a prerequisite for reverse transcription and transition to PIC formation [6]. Binding of the Trim-cyclophilin restriction system to capsid, as well as the CAp24 mutations that affect capsid stability, effectively invoke defects in reverse transcription, nuclear import or integration [55], [56]. Therefore we propose that reverse transcription and disruption of the nucleocapsid might be coupled to capsid uncoating in a process that requires further exploration. Such changes may also be linked to the trafficking of the HIV-1 genome during cellular infection [5], [57], in order to expose key components to the required cellular partners, i.e., actin, microtubule-associated molecular motors and nuclear import machinery [58]. Further *in vitro* reconstitution experiments using direct visualization will help to understand better the related molecular mechanisms that control the plasticity and the movement of the HIV-1 nucleoprotein architecture during viral maturation as well as in the early steps of cellular infection.

Finally, we believe that this remodelling strategy of nucleoprotein architecture is a characteristic that may be employed by most, if not all mobile genetic elements to optimize their capability for propagation within host cells and thus to ensure their survival. Virally encoded proteases, RNA/DNA-polymerases and RNA/DNA-helicases should be carefully examined for their possible contribution to this strategy.

## Methods

### Protein, DNA and reagents

Recombinant HIV-1 PR and RT were prepared as described [59], [60]. HIV-1 PR was titrated fluorometrically [61]. RT concentration was determined using an extinction coefficient at 280 nm of 260,450 M^−1^cm^−1^. Recombinant HIV-1 wild-type NCp(1-55) (i.e. NCp7) and NCp15 (based on sequences from GenBank, accession number **AF324493)** were expressed and purified as described [62]–[64]. The p6 peptide was provided by S. Bouaziz (Université Paris 5). Mo-MuLV NCp10 was expressed and purified essentially as described above for HIV-1 NCp7. Alternatively, synthetic Mo-MuLV NCp10 was obtained as a generous gift from J. L. Darlix (ENS, Lyon). *AlwNI*, *DraI* and Mo-MuLV RT enzymes were purchased from New England Biolabs (Ipswich, MA). The circular 3,352 nt single-stranded DNA, that contains the HIV-Bru cPPT-CTS sequence, as well as its plasmid counterpart, were prepared as described [30]. The 17 mer-oligonucleotide ODN 17 (DNA, 5′-TTGGGGGGTACAGTGCA) used for the G4-DNA and as a primer for reverse transcription was purchased from Eurogentec (Seraing, Belgium). DNA concentrations were measured in a Genequant microspectrophotometer (APBiotech). The 1-415 RNA fragment corresponding to the HIV-1 leader region was a generous gift from J. L. Darlix (ENS, Lyon). All other reagents used in this study were of the highest grade available and were purchased from established manufacturers.

### NCp15 proteolysis

NCp15 (2 µM) was first incubated with nucleic-acids (ssDNA, 2.5 nM; G4-DNA, 500 nM; RNA, 40 nM; dsDNA, 2 nM) for 10 min. at 37°C in 10 µl of a PR buffer (0.02% [w/v] KCl, 0.012% KH_2_PO_4_, 0.091% Na_2_HPO_4_, pH 6.6) containing 100 mM NaCl. Next, PR equilibrated in its reaction buffer was added for 2 h at 30°C. The reaction was stopped by the addition of an equal volume of SDS-PAGE loading buffer. Reaction products were heated to 100°C prior to Tris-Tricine SDS-PAGE on an 8–16% gradient gel followed by silver staining (Invitrogen). Purified NCp7, NCp15 and p6 proteins were used as migration controls.

### G4-DNA Electrophoretic Mobility Shift Assay (EMSA)

^33^P-labelled parallel G-quadruplex DNA was prepared by interstrand association of the ODN 17 (with 200 mM KCl at 40°C for 30 h) prior to radiolabelling as described [28]. The standard G4-DNA binding assay (10 µl) was performed as described [29]. The fraction of NCp-bound DNA was determined as the ratio of bound DNA (all the shifted bands for NCp7) to the total number of counts in each lane. The apparent equilibrium dissociation constant (K_dapp_) was estimated via the protein concentration necessary for half maximal DNA binding.

### DNA synthesis

The 3,352-nt circular ssDNA template was hybridized to ODN 17. The polymerase reaction was carried out in the RT buffer (50 mM Tris-acetate pH 7.8, 50 mM sodium acetate, 6 mM magnesium acetate and 0.5 mM DTT) at 37°C. For analysis by agarose gel electrophoresis, DNA products were heated to 70°C for 10 min. in the presence of 1% (w/v) SDS and 20 mM EDTA, (see [30] for more details). Samples of DNA produced in the presence of NCp7 were also centrifuged for 10 min at 9000×*g* at 4°C and the upper half volume of each sample was separated very carefully before heat treatment and electrophoresis.

### Transmission Electron Microscopy

EM observations were performed with a positive staining procedure as described previously [30], [65], [66]. 5 µl-aliquots of NCp/DNA complexes (DNA concentration of 2.5–5 nM) were diluted 5-fold in the reaction buffer without DTT, deposited onto the EM grid, rinsed with an aqueous uranyl acetate solution (2% w/v) and dried. This deposition procedure rapidly adsorbed DNA molecules onto the carbon film with no significant loss of three-dimensional information [67]. To avoid precipitation of phosphate buffer with uranyl acetate, NCp15 proteolysis by PR was performed in 50 mM sodium-acetate (pH 6.0) and 4 mM magnesium-acetate. Aliquots were then diluted 5-fold in 50 mM HEPES pH 7.0 before their deposition onto the grid. Negative films of NCp-DNA complexes were captured for final magnifications of up to 85,000×, and then converted to digital images with a high-resolution scanner (LaCie BlueScan).

## Supporting Information

Animation S1 An animated model of HIV-1 reverse transcription. In order to better understand the overall process of HIV reverse transcription, an animated model is available here for downloading. Animation S1 is a MOV file for viewing with Apple Quicktime and Animation S2 is a SWF file for viewing with the Flash plugin of Microsoft Internet Explorer or Mozilla Firefox web browsers. Note that with the MOV file (S2) the space bar is available to pause and to start the animation. This animation is derived from the classical model of reverse transcription [A. Telesnitsky and S. P. Goff, Reverse transcriptase and the generation of retroviral DNA, in Retrovirus, J. M. Coffin, S. H. Hughes and H. E. Varmus Eds, (1997) CSHL Press]. For clarity, this animation represents an idealized process with a figure-of-eight-shaped nucleic-acid template. Attention has been focused on the following points: the three primers (tRNALys3, 3′ppt and cppt) are oversized for clarity; the two RNA ends (RU5 and U3R) are in close proximity throughout reverse transcription, in order to show a continuous process at the time of the strand transfers; the RT-associated RNAseH is shown with a high activity for digestion of the RNA following minus strand DNA elongation. The two polypurine tracts are presented as the two exclusive primers for plus strand DNA initiation, whereas the remaining RNA fragments are proposed to be displaced by the two translocating RT molecules during minus strand DNA elongation. The central DNA flap is viewed as a fluctuating structure, the function of which still needs to be elucidated. The presentation of the timing for plus strand DNA termination in combination with the central flap synthesis and LTR duplication seems the most representative to the authors. This model does not exclude alternative pathways, especially considering the dimeric nature of the initial RNA template.
(0.13 MB MOV)

Animation S2 See Legend of Animation S1.
(0.13 MB SWF)

Figure S1 Differences in DNA synthesis depending on the origin of NCp and RT. (A) Comparison of the effects of HIV-1 NCp7, NCp9 and NCp12-53 vs. Mo-MuLV NCp10 (3 µM) on DNA synthesis catalysed by HIV-1 RT (50 nM) and analysed after 10 and 40 min. incubation. Samples were micro-sedimented before electrophoresis to evaluate the level of DNA aggregation. (B) TEM of DNA synthesis after 40 min. of incubation with HIV-1 RT and NCp9. (C) Comparison of DNA synthesis by HIV-1 RT (200 nM) and Mo-MuLV RT (200 U) after 20 and 60 min. in the absence or presence of HIV-1 NCp7 or Mo-MuLV NCp10 (3 µM) respectively, without micro-sedimentation of the samples. (D) TEM visualization of DNA synthesis with Mo-MuLV RT and NCp10. The scale bars correspond to 250 nm.
(5.60 MB TIF)

Dataset S1 Comments for Figure S1
(0.02 MB DOC)
