# Supplementary figures and images for: HIV-1 Protease and Reverse Transcriptase Control the Architecture of Their Nucleocapsid Partner
Source: PLoS One. 2007 Aug 22;2(8):e669. doi: 10.1371/journal.pone.0000669 (PMC1940317; doi:10.1371/journal.pone.0000669)

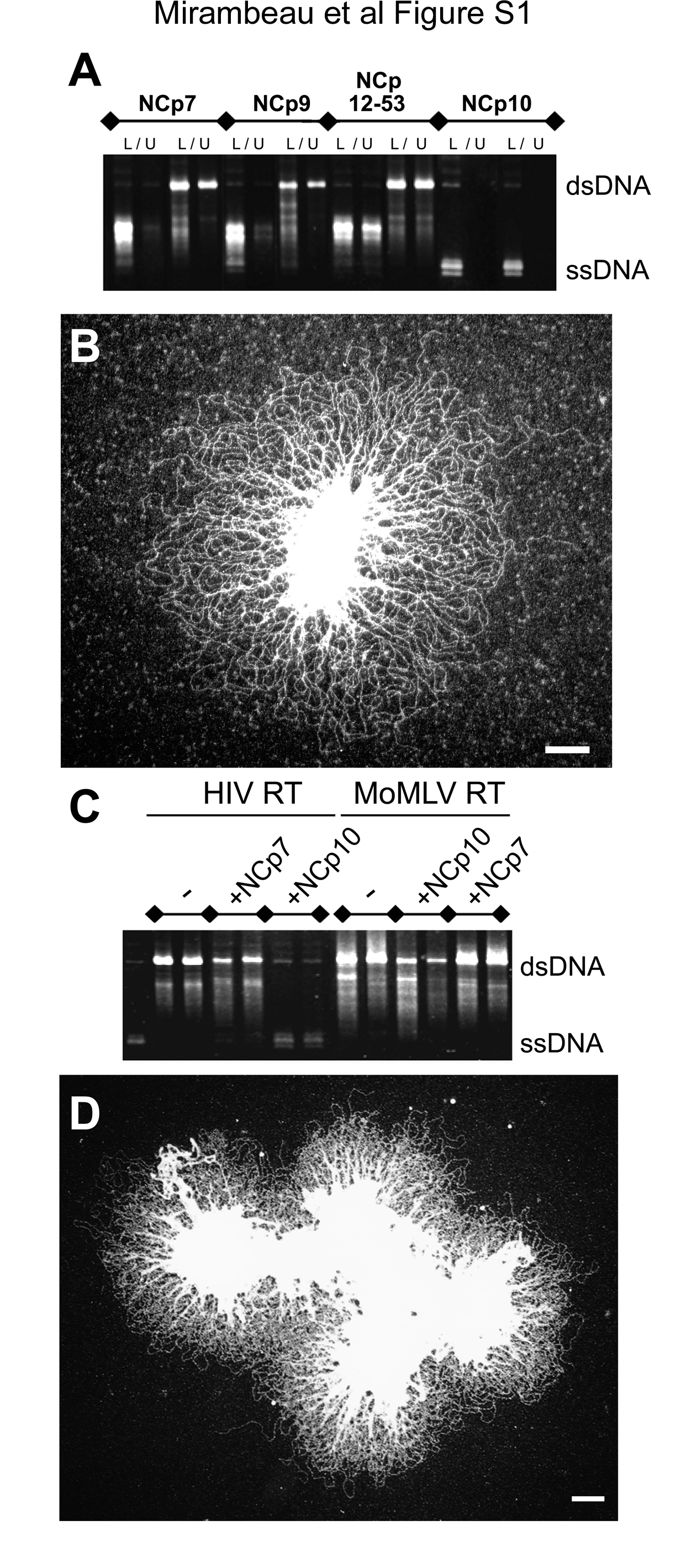

Supplement: Figure S1 — Differences in DNA synthesis depending on the origin of NCp and RT. (A) Comparison of the effects of HIV-1 NCp7, NCp9 and NCp12-53 vs. Mo-MuLV NCp10 (3 µM) on DNA synthesis catalysed by HIV-1 RT (50 nM) and analysed after 10 and 40 min. incubation. Samples were micro-sedimented before electrophoresis to evaluate the level of DNA aggregation. (B) TEM of DNA synthesis after 40 min. of incubation with HIV-1 RT and NCp9. (C) Comparison of DNA synthesis by HIV-1 RT (200 nM) and Mo-MuLV RT (200 U) after 20 and 60 min. in the absence or presence of HIV-1 NCp7 or Mo-MuLV NCp10 (3 µM) respectively, without micro-sedimentation of the samples. (D) TEM visualization of DNA synthesis with Mo-MuLV RT and NCp10. The scale bars correspond to 250 nm. (5.60 MB TIF) [file pone.0000669.s003.tif]
