# Supplementary material for: HIV-1 Protease and Reverse Transcriptase Control the Architecture of Their Nucleocapsid Partner
Source: PLoS One. 2007 Aug 22;2(8):e669. doi: 10.1371/journal.pone.0000669 (PMC1940317; doi:10.1371/journal.pone.0000669)
Supplement: Dataset S1 — Comments for Figure S1 (0.02 MB DOC) [file pone.0000669.s004.doc]

**Dataset S1: NCp-ssDNA co-aggregation followed by nascent dsDNA extrusion characterizes the wild-type HIV-1 NCp7-RT pair.**

In addition to the mature 55 aa-form (NCp7), three peptides have been tested under the same saturating conditions in our RT assay (Figure S1): NCp9, a 72 aa intermediate form; NCp12-53, a 41 aa-form, which lacks 12 aa and 2 aa from the N- and C-termini respectively; and NCp10, which is the corresponding mature NCp from the Moloney-murine leukemia virus (Mo-MuLV). With NCp9, results are basically the same as those obtained with NCp7 (Figure S1, A, B). ssDNA was fully aggregated, and upon DNA synthesis the dsDNA seceded from the aggregates. However, relaxation of the aggregates and extrusion of the dsDNA only appeared at the periphery, whereas they also occurred extensively in the interior of the aggregates with NCp55 (compare Figures S1, B and. 4B). With the NCp12-53, very limited aggregation of ssDNA was observed. DNA synthesis proceeded efficiently, however, neither ssDNA nor dsDNA pelleted in the microsedimentation assay (Figure S1, A), whereas a limited number of DNA circles remained associated (TEM, not shown). With NCp10, RT was totally inhibited, while ssDNA remained fully aggregated (Figure S1, A). DNA products from HIV-1 and Mo-MuLV RT enzymes were also compared in our RT assay after 20 and 60 min. incubation in the absence of NCp, or in the presence of saturating concentrations of either HIV-1 NCp7 or Mo-MuLV NCp10 (Figure S1, C). The concentration of HIV-1 RT was increased fourfold (200 nM). An equivalent amount of Mo-MuLV RT (200 U) was used to perform DNA synthesis along the DNA circles within the same time frame as HIV-1 RT. Regardless of this change, there was still complete inhibition of HIV-1 RT by the Mo-MuLV NCp10. Conversely, DNA synthesis catalysed by Mo-MuLV RT led to the accumulation of the typical discrete bands on an agarose gel, referred to as dsDNA circles, while a slower migrating smear was also visible, corresponding to the sub-population engaged in strand-displacement synthesis. Even though Mo-MuLV RT appeared more efficient than HIV-1 RT in promoting strand-displacement synthesis in the cPPT-CTS locus, a significant pause occurred after one round of DNA synthesis. Adding Mo-MuLV NCp10 maintained an efficient reaction. However, DNA products were partially lost because of their retention in the wells of the agarose gel. This suggested that the aggregates formed during the reaction were more stable with NCp10 than with NCp7. By applying TEM visualization (Figure S1, D), the dsDNA produced by Mo-MuLV RT appeared to co-aggregate extensively with NCp10, presumably with the prominent ssDNA tails centered toward the more dense portions of the aggregates. These structures were most likely to be due to the efficient strand displacement activity of Mo-MuLV RT that was not arrested at the central termination site. This conclusion is compatible with the finding of massive co-aggregates obtained when Mo-MuLV NCp10 was mixed with similar concentrations of ssDNA and dsDNA (Mirambeau et al., 2006, *J Mol Biol*, 364, 496-511). Finally, Mo-MuLV RT, in the presence of HIV-1 NCp7, generated a slightly different secondary pausing profile for simple DNA elongation compared to Mo-MuLV RT alone, and in addition all the DNA circles entered into the gel (Figures S1, C). Therefore, HIV-1 NCp appeared to be compatible with Mo-MuLV RT, although Mo-MuLV NCp was not with HIV-1 RT.
